# Relationship between screw sagittal angle and stress on endplate of adjacent segments after anterior cervical corpectomy and fusion with internal fixation: a Chinese finite element study

**DOI:** 10.1186/s12893-017-0305-z

**Published:** 2017-12-01

**Authors:** Yu Zhang, Yibo Tang, Hongxing Shen

**Affiliations:** 1 Department of Medicine, Chinese People’s Liberation Army General Hospital and Hainan Branch, Sanya, China; 2 Department of Orthopaedics, Second Military Medical University of Chinese People’s Liberation Army and Changhai Hospital, Shanghai, China

**Keywords:** Adjacent segment disease, Anterior cervical corpectomy and fusion with internal fixation, Finite element model, Screw sagittal angle

## Abstract

**Background:**

In order to reduce the incidence of adjacent segment disease (ASD), the current study was designed to establish Chinese finite element models of normal 3rd~7th cervical vertebrae (C3-C7) and anterior cervical corpectomy and fusion (ACCF) with internal fixation, and analyze the influence of screw sagittal angle (SSA) on stress on endplate of adjacent cervical segments.

**Methods:**

Mimics 8.1 and Abaqus/CAE 6.10 softwares were adopted to establish finite element models.

**Results:**

For C4 superior endplate and C6 inferior endplate, their anterior areas had the maximum stress in anteflexion position, and their posterior areas had the maximum stress in posterior extension position. As SSA increased, the stress reduced. With an increase of 10° in SSA, the stress on anterior areas of C4 superior endplate and C6 inferior endplate reduced by 12.67% and 7.99% in anteflexion position, respectively. With an increase of 10° in SSA, the stress on posterior areas of C4 superior endplate and C6 inferior endplate reduced by 9.68% and 10.22% in posterior extension position, respectively.

**Conclusions:**

The current study established Chinese finite element models of normal C3-C7 and ACCF with internal fixation, and demonstrated that as SSA increased, the stress on endplate of adjacent cervical segments decreased. In clinical surgery, increased SSA is able to play important role in protecting the adjacent cervical segments and reducing the incidence of ASD.

## Background

Anterior cervical corpectomy and fusion (ACCF) is a common treatment for cervical spondylosis and has obvious curative effect for cervical degenerative disease and cervical radiculopathy. However, as an inevitable complication after ACCF, adjacent segment disease (ASD) has a prevalence of 7%–17% and leads to a secondary surgery rate of more than 15% [1–3]. Elevated stress on vertebral body and internal pressure of vertebral disc accelerate the degeneration of adjacent cervical segments [4–6]. Mozammil Hussain and his colleagues have suggested that screw sagittal angle (SSA) might affect the stress on vertebral body and disc and the secondary surgery rate caused by ASD [7]. However, studies discussing the relationship of SSA with stress on endplate and development of ASD are scarce, especially in China.

Previous methods exploring the biomechanics of human body, such as animal model, physical model and cadaver model, all have unavoidable drawbacks. Along with the improvement in finite element theory and computer system, finite element method has been widely used in analyzing the biomechanics of human body. Accordingly, finite element model has been put into application and shown its advantage in the studies on cervical spine [8, 9]. Finite element model is able to not only realistically simulate the structure of cervical vertebral body, vertebral disc and ligament, but also scientifically analyze the stress on the structure [10]. However, finite element model is not widely used in Chinese scientific research and clinical work.

The aim of the current study was to establish Chinese finite element models of normal 3rd~7th cervical vertebrae (C3-C7) and ACCF with internal fixation, and analyze the influence of SSA on stress on endplate of adjacent cervical segments.

## Methods

### Model building

**Fig. 1:**
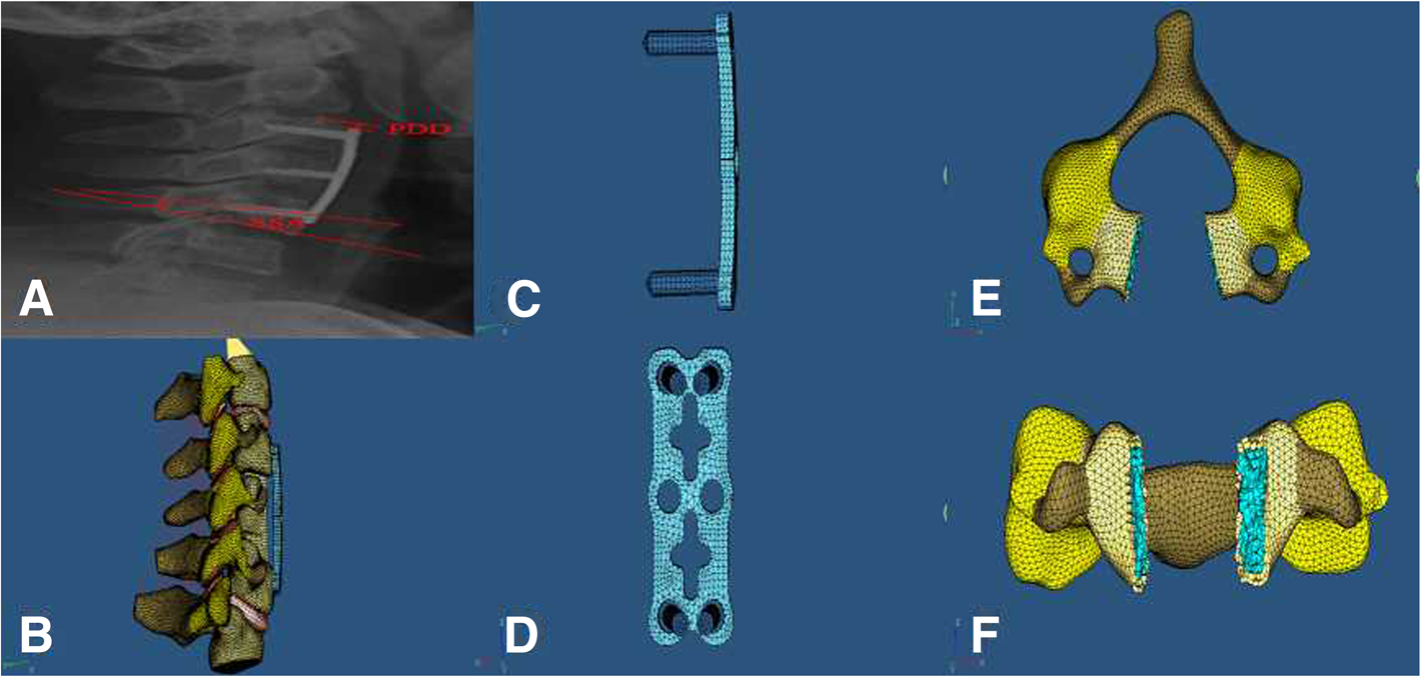
measurement of screw sagittal angle (SSA) and plate disc distance (PDD; **a**); finite element model of the 5th cervical vertebrae with anterior cervical corpectomy and fusion with internal fixation (lateral view; **b**); finite element model of steel plate and screw (lateral view; **c**); finite element model of steel plate and screw (inside view; **d**); finite element model of the 5th cervical vertebrae with anterior cervical corpectomy (superior view; **e**); finite element model of the 5th cervical vertebrae with anterior cervical corpectomy (anterior view; **f**)

Two-dimensional transversal images obtained from computerized tomography scan (scanning condition: 120KV, 125 mA, layer thickness 0.625 mm, interlayer spacing 0 mm) of Chinese normal male volunteer without disease related to cervical spine were imported into Mimics 8.1 software (Materialise, Leuven, Belgium) to compute the coronal and sagittal images. Images of different tissues were extracted based on grey threshold. Three-dimensional image was imported into Abaqus/CAE 6.10 software (Dassault Systemes, Velizy-Villacoublay, France) to form the geometric model of C3-C7. Boundary conditions and loading states were in the following: lower surface of C7 was completely fixed, and unfixed C3 received the load of 73.6 N with an additional torque of 1.8 Nm, thus making the model able to have anteflexion and posterior extension movement. Parameters of tissues and materials including elastic modulus and poisson’s ratio were input into model (Table 1). In established finite element model of normal C3-C7, range of motion for each vertebral disc was measured when having anteflexion and posterior extension movement, and then compared with history data (John study). Finite element models of steel plate and screw were established using the preprocessor of Abaqus/CAE 6.10 software. Anterior longitudinal ligament, C4/5 and C5/6 vertebral disc, C5 vertebral body and posterior longitudinal ligament were orderly removed from finite element model of normal C3-C7. Bone graft was medially implanted with a cross-sectional area accounting for half of removed vertebral body. Steel plate was fixed on C4 and C6 vertebral body with two 18 mm screws on the top and another two screws on the bottom. SSA was an angle between screw and adjacent endplate in sagittal position, and set as three kinds including 0°, 5° and 10° (Fig. 1). Vertebral body, bone graft and steel plate were closely contacted without sliding and deformation.

**Table 1:** Features of tissues and materials

Tissues and materials	Elastic modulus(MPa)	Poisson’s ratio
Cortical bone	10,000.00	0.29
Cancellous bone	100.00	0.29
Posterior structure	3500.00	0.29
Endplate	5.00	0.40
Annulus fibrosus	2.50	0.40
Nucleus pulposus	1.50	0.49
Anterior longitudinal ligament	15	–
Posterior longitudinal ligament	10	–
Interspinal ligament	2	–
Ligamentum flavum	5	–
Capsular ligament	7	–
Steel plate	11,0000.00	0.30
Screw	11,0000.00	0.30
Bone graft	3500.00	0.30

### Data analysis

Model was dealt with Abaqus/CAE solver, and then analyzed by Abaqus/CAE postprocessor. Endplate was divided into anterior, posterior, left and right areas, and steel plate was divided into upper, middle and lower areas. Stress on these areas was recorded and shown as Mises stress (MPa).

## Results

Established finite element model of normal C3-C7 included all the tissues including cortical bone, cancellous bone, posterior structure, endplate, annulus fibrosus, nucleus pulposus, anterior longitudinal ligament, posterior longitudinal ligament, interspinal ligament, ligamentum flavum and capsular ligament, and had a total of 337,188 nodes and 130,603 elements (Table 1). Range of motion for each vertebral disc was similar to history data (Table 2).

**Table 2:** Range of motion (°) for each vertebral disc

Studies	C3/4	C4/5	C5/6	C6/7
John study	5.13	6.48	6.04	6.54
Current study	5.04	5.92	5.53	6.15

For C4 superior endplate and C6 inferior endplate, their anterior areas had the maximum stress in anteflexion position, and their posterior areas had the maximum stress in posterior extension position (Table 3). As SSA increased, the stress reduced. With an increase of 10° in SSA, the stress on anterior areas of C4 superior endplate and C6 inferior endplate reduced by 12.67% and 7.99% in anteflexion position, respectively. With an increase of 10° in SSA, the stress on posterior areas of C4 superior endplate and C6 inferior endplate reduced by 9.68% and 10.22% in posterior extension position, respectively.

**Table 3:** Stress (MPa) on endplate and steel plate

Tissues and materials	Areas	Anteflexion			Posterior extension		
		0°	5°	10°	0°	5°	10°
C4 superior endplate	Anterior	8.21	7.52	7.17	1.30	1.31	1.30
	Posterior	2.34	2.21	2.12	8.99	8.35	8.12
	Left	1.27	1.39	1.27	1.54	1.70	1.86
	Right	1.48	1.45	1.56	1.24	1.16	1.17
C6 inferior endplate	Anterior	4.13	4.27	3.80	1.64	1.57	1.52
	Posterior	3.17	2.92	2.78	3.62	3.46	3.25
	Left	2.76	2.52	2.28	2.71	2.56	2.51
	Right	3.04	3.13	3.10	3.50	3.31	3.30
Steel plate	Upper	3.31	3.78	3.84	2.15	3.01	3.45
	Middle	16.20	17.31	19.37	12.24	12.56	13.05
	Lower	2.43	2.48	2.53	2.36	2.53	2.63

Middle area of steel plate had the maximum stress in anteflexion and posterior extension position (Table 3). As SSA increased, the stress rose. With an increase of 10° in SSA, the stress on middle area of steel plate rose by 19.57% in anteflexion position and 6.62% in posterior extension position.

## Discussion

Previous methods exploring the biomechanics of human body, such as animal model, physical model and cadaver model, are able to simulate the structure of cervical spine, but they all have unavoidable drawbacks. Animal model, such as pig or sheep, has absolutely different stress mode compared with human walking upright. Material characteristics of physical model are different from those of human body. Cadaver model is not only rare and expensive, but also lack of biological change under the physiological condition.

As finite element theory and computer system improve, finite element method has been widely applied in analyzing the biomechanics of human body. Accordingly, finite element model has been gradually used and continually improved in the studies on cervical spine [8, 9]. Finite element model is able to simulate the anatomical structure and material characteristics of cervical spine, imitate the complicated physiological condition, reflect the stress on the arbitrary area of cervical spine or the whole cervical spine, and be applied repeatedly due to its stability [10]. However, finite element model is not widely used in Chinese scientific research and clinical work.

The current study adopted Mimics 8.1 and Abaqus/CAE 6.10 softwares to establish finite element models of normal C3-C7 and ACCF with internal fixation. Abaqus/CAE 6.10 software is mainly focused on the research in structural mechanics and related fields, and able to analyze more common non-linear problems involving material non-linear, geometric non-linear, and state non-linear fields. Applying the Abaqus/CAE 6.10 software is able to get the results in line with actual condition. Established finite element models based on thin slice CT scan in the current study were identical with basic structure and functional status of cervical spine. Moreover, precision of finite element method depends on the numbers of nodes and elements, and finite element model of normal C3-C7 in the current study had a total of 337,188 nodes and 130,603 elements showing good accuracy. Range of motion for each vertebral disc in this model was similar to history data, confirming its validity and availability for modeling ACCF with internal fixation.

ADS is a new onset cervical myelopathy or cervical radiculopathy of adjacent segments after ACCF [4–6]. Pathogenesis of ADS remains unclear, and the widely accepted one is biomechanics [7–9]. ACCF changes the local and overall structure and biomechanics of cervical spine and results in excessive stress on adjacent cervical segments beyond the scope of normal physiology [11]. Mozammil Hussain and his colleagues have proposed that increased SSA might reduce the stress on adjacent cervical segments and avoid the development of ASD after ACCF with internal fixation [10]. However, there are few studies focusing on the relationship of SSA with stress on endplate and development of ASD, especially in China. The current study showed that as SSA increased, the stress on C4 superior endplate and C6 inferior endplate decreased accompanying with raised stress on steel plate. In other words, a larger SSA is able to protect the endplate and vertebral disc of adjacent cervical segments from excessive stress, delay the degeneration process of vertebral disc, and reduce the incidence of ASD.

## Conclusions

The current study established Chinese finite element models of normal C3-C7 and ACCF with internal fixation, and demonstrated that as SSA increased, the stress on endplate of adjacent cervical segments decreased. In clinical surgery, increased SSA is able to play important role in protecting the adjacent cervical segments and reducing the incidence of ASD.

## Abbreviations

ACCF: Anterior cervical corpectomy and fusion
ASD: Adjacent segment disease
C3-C7: The 3rd~7th cervical vertebrae
SSA: Screw sagittal angle
